# Species characteristics of felids and canids, and the number of articles published for each species between 2013 and 2017

**DOI:** 10.1016/j.dib.2018.09.132

**Published:** 2018-10-03

**Authors:** L. Tensen

**Affiliations:** The Centre for Ecological Genomics and Wildlife Conservation, Department of Zoology, University of Johannesburg, Auckland Park Campus, 2006, South Africa

## Abstract

The data presented are related to the research article entitled “Biases in wildlife and conservation research, using felids and canids as a case study” available at https://doi.org/10.1016/j.gecco.2018.e00423. This data article lists species characteristics of two families of the order Carnivora, the Felidae and Canidae, and quantitatively categorizes research output for each species. The species characteristics that were included in the dataset are body size (in kg), geographic range size, IUCN species status, population trend, likelihood of being a keystone species, number of species per genus, the Evolutionary Distinctiveness (ED) score, and the Evolutionary Distinct and Globally Endangered (EDGE) score. All scientific articles that were published on felid and canid species between 2013 and 2017 were listed and subdivided into the following research topics: (1) ecology and behaviour, (2) conservation and wildlife management, (3) anatomy and physiology, (4) diseases and other health issues, (5) captive housing and artificial reproduction, (6) genetic diversity and phylogenetic structure, and (7) taxonomy and palaeoecology. All the data is made publically available.

**Specifications table**

**Table:** 

Subject area	*Biology*
More specific subject area	*Conservation management*
Type of data	*Table, Figure*
How data was acquired	*The data was obtained from online literature searching engines Scopus, EBSCO and Google Scholar*
Data format	*Raw and partially analysed*
Experimental factors	*None*
Experimental features	*Quantitative data analysis*
Data source location	*Not applicable*
Data accessibility	*Data is presented in this article and publically available for educational, commercial, or scientific purposes.*

**Value of the data**

• This data can be used to find trends and gaps in carnivore research.
• This data can assist in setting prioritization schemes for conservation.
• This data can highlight biases in wildlife and conservation research.

## 1 Data

It is important find biases in wildlife research to better allocate conservation funds in the future [1]. For instance, there is a research-implementation gap in scientific research with regards to species conservation [2], [3], [4]. Certain species are being studied considerably more often than other species [5], [6], and research is not yet focussed on taxa that need it the most [7]. Preferably, wildlife biologists should attempt to focus on species that are endangered, have a limited geographic range, fill a keystone role in the ecosystem, or are taxonomically distinct [1], [2], [4]. This article lists species characteristics of two families of the order Carnivora: the Felidae (hereafter felids) and Canidae (hereafter canids), and quantitatively categorizes research output for each species.

This article includes 37 felid species and 36 canid species. Body size was based on average weight (in kg) derived from [8] for felid species and [9] for canid species (Table 1). Body weight ranged from 1.8 to 173 kg in felids, and 1 to 39 kg in canids.

**Table 1:** All felid and canid species included in this dataset and their average body weight (in kg).

**Felidae species**			**Canidae species**		
**Species name**	**Scientific name**	**Weight**	**Species name**	**Scientific name**	**Weight**
Cheetah	*Acinonyx jubatus*	38.7	Short eared dog	*Atelocynus microtis*	9.5
African golden cat	*Caracal aurata*	9.1	Side-striped jackal	*Canis adustus*	8.8
Caracal	*Caracal caracal*	11.5	African golden wolf	*Canis anthus*	11
Bay cat	*Catopuma badia*	2.3	Golden jackal	*Canis aureus*	8.1
Asiatic golden cat	*Catopuma temminckii*	10.7	Coyote	*Canis latrans*	10.9
Jungle cat	*Felis chaus*	6.6	Grey wolf	*Canis lupus*	39
Sand cat	*Felis margarita*	2.5	Black-backed jackal	*Canis mesomelas*	7.8
Black footed cat	*Felis nigripes*	1.6	Red wolf	*Canis rufus*	26.4
Wild cat	*Felis silvestris*	4.3	Ethiopian wolf	*Canis simensis*	14.5
Ocelot	*Leopardus pardalis*	11.7	Crab-eating fox	*Cerdocyon thous*	5.7
Southern tigrina	*Leopardus guttulus*	2.1	Maned wolf	*Chrysocyon brachyurus*	25
Oncilla	*Leopardus tigrinus*	2.4	Dhole	*Cuon alpinus*	15.8
Margay	*Leopardus wiedii*	3.3	Culpeo	*Lycalopex culpaeus*	9.8
Pampas cat	*Leopardus colocolo*	4	Darwin׳s fox	*Lycalopex fulvipes*	3.1
Geoffroy׳s cat	*Leopardus geoffroyi*	5.1	South American gray fox	*Lycalopex griseus*	3.7
Kodkod	*Leopardus guigna*	1.6	Pampas fox	*Lycalopex gymnocercus*	4.4
Andean mountain cat	*Leopardus jacobita*	4.5	Sechura fox	*Lycalopex sechurae*	3.6
Serval	*Leptailurus serval*	9.9	Hoary fox	*Lycalopex vetulus*	3.4
Canada lynx	*Lynx canadensis*	9.7	African wild dog	*Lycaon pictus*	26
Eurasian lynx	*Lynx lynx*	18.5	Raccoon dog	*Nyctereutes procyonoides*	4.5
Iberian lynx	*Lynx pardinus*	11.1	Bat-eared fox	*Otocyon megalotis*	4.1
Bobcat	*Lynx rufus*	7.8	Bush dog	*Speothos venaticus*	6.5
Clouded leopard	*Neofelis nebulosa*	14.8	Grey fox	*Urocyon cinereoargenteus*	3.7
Sunda clouded leopard	*Neofelis diardi*	15.5	Island fox	*Urocyon littoralis*	1.9
Manul	*Otocolobus manul*	4.1	Bengal fox	*Vulpes bengalensis*	2.4
Lion	*Panthera leo*	146.3	Blandford׳s fox	*Vulpes cana*	1
Jaguar	*Panthera onca*	85.7	Cape fox	*Vulpes chama*	2.7
Leopard	*Panthera pardus*	41.8	Corsac fox	*Vulpes corsac*	2.4
Tiger	*Panthera tigris*	173	Tibetan fox	*Vulpes ferrilata*	3.8
Snow leopard	*Panthera uncia*	37.6	Arctic fox	*Vulpes lagopus*	3.4
Marbled cat	*Pardofelis marmorata*	3.1	Kit fox	*Vulpes macrotis*	2.1
Leopard cat	*Prionailurus bengalensis*	2.6	Pallid fox	*Vulpes pallida*	2.8
Flat-headed cat	*Prionailurus planiceps*	1.8	Rüppell׳s fox	*Vulpes rueppellii*	1.5
Rusty-spotted cat	*Prionailurus rubiginosus*	0.9	Swift fox	*Vulpes velox*	2.1
Fishing cat	*Prionailurus viverrinus*	9.3	Red fox	*Vulpes vulpes*	5.8
Puma	*Puma concolor*	44.8	Fennec fox	*Vulpes zerda*	1.5
Jaguarundi	*Puma yagouaroundi*	4.9			

**Fig. 1:**
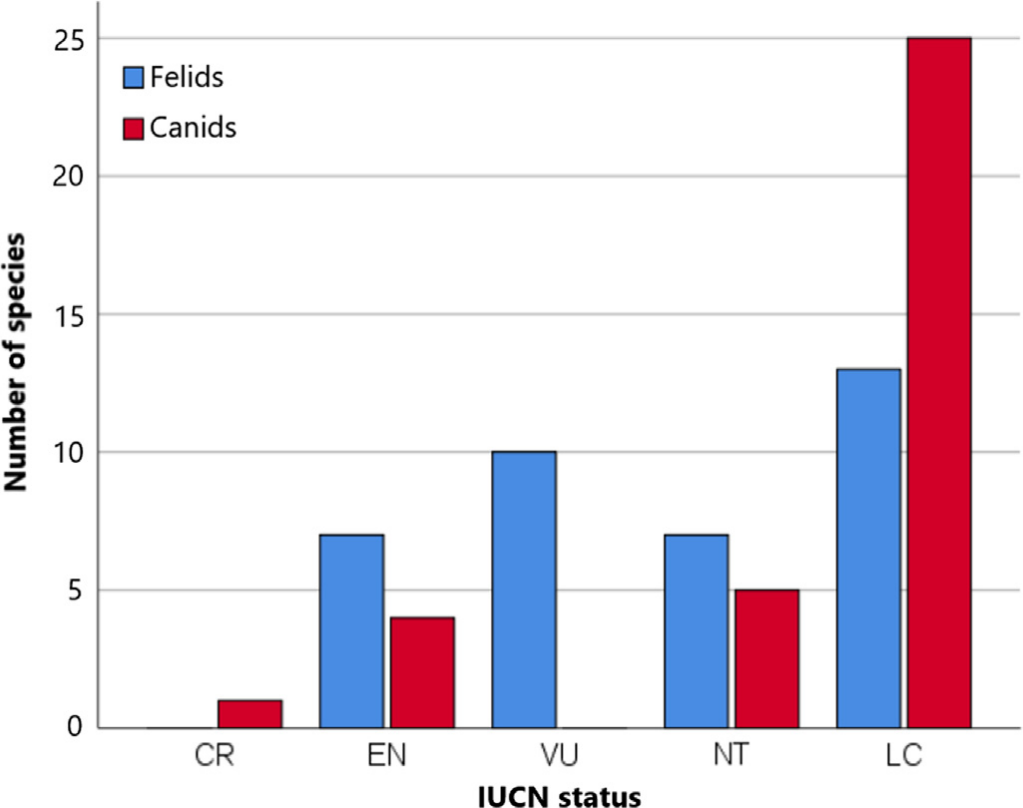
IUCN status of felid and canid species.

**Fig. 2:**
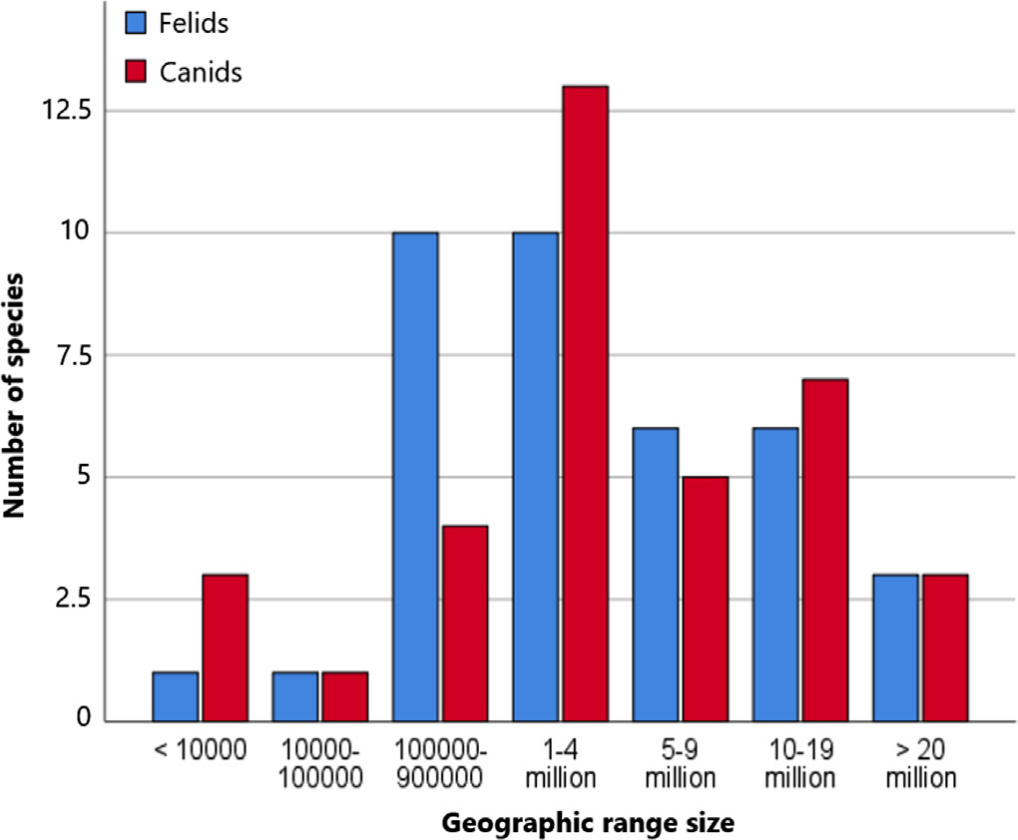
Geographic range size of felid and canid species.

The conservation status, population trend and geographic range size were listed for each species (Table 2) and based on the IUCN (International Union for Conservation of Nature and Natural Resources) Red List of Threatened Species [10]. For IUCN status, species with a higher risk of extinction are ranked in higher categories, from Data Deficient (DD), Least Concern (LC) to Near Threatened (NT), Vulnerable (VU), Endangered (EN), and Critically Endangered (CR). Most species are of Least Concern, and felid species are more often threatened with extinction than canids (Fig. 1). Population trend is either unknown, decreasing, stable, or increasing. Geographic range size was based on distribution maps provided by the IUCN and divided into seven categories for the purpose of this data overview: (1) < 10,000 km^2^; (2) 10,000–100,000 km^2^; (3) 100,000–900,000 km^2^; (4) 1–4 million km^2^; (5) 5–9 million km^2^; (6) 10–19 million km^2^; and (7) > 20 million km^2^. Most species had a geographic range size of 1 to 4 million km^2^ (Fig. 2).

**Table 2:** The IUCN status, population trend and geographic range size for felid and canid species. IUCN status was Least Concern (LC), Near Threatened (NT), Vulnerable (VU), Endangered (EN), or Critically Endangered (CR). Geographic range size was (1) < 10,000 km^2^, (2) 10,000–100,000 km^2^, (3) 100,000–900,000 km^2^, (4) 1–4 million km^2^, (5) 5–9 million km^2^, (6) 10–19 million km^2^, or (7) > 20 million km^2^.

**Felidae species**				**Canidae species**			
**Species name**	**IUCN status**	**Population trend**	**Range size**	**Species name**	**IUCN status**	**Population trend**	**Range size**
Cheetah	EN	stable	4	Short eared dog	NT	decreasing	4
African golden cat	VU	decreasing	4	Side-striped jackal	LC	stable	6
Caracal	LC	unknown	6	African golden wolf	not listed	unknown	3
Bay cat	EN	decreasing	3	Golden jackal	LC	increasing	7
Asiatic golden cat	NT	decreasing	3	Coyote	LC	increasing	6
Jungle cat	LC	decreasing	5	Grey wolf	LC	stable	7
Sand cat	LC	unknown	3	Black-backed jackal	LC	stable	5
Black footed cat	VU	decreasing	3	Red wolf	CE	increasing	1
Wild cat	LC	decreasing	7	Ethiopian wolf	EN	decreasing	1
Ocelot	LC	decreasing	6	Crab-eating fox	LC	stable	5
Southern tigrina	VU	decreasing	4	Maned wolf	NT	unknown	4
Oncilla	VU	decreasing	5	Dhole	EN	decreasing	4
Margay	NT	decreasing	6	Culpeo	LC	stable	4
Pampas cat	NT	decreasing	4	Darwin׳s fox	EN	decreasing	2
Geoffroy׳s cat	LC	stable	4	South American gray fox	LC	stable	3
Kodkod	VU	decreasing	3	Pampas fox	LC	stable	4
Andean mountain cat	EN	decreasing	3	Sechura fox	NT	unknown	3
Serval	LC	stable	6	Hoary fox	LC	unknown	4
Canada lynx	LC	stable	5	African wild dog	EN	decreasing	4
Eurasian lynx	LC	stable	7	Raccoon dog	LC	stable	5
Iberian lynx	EN	increasing	1	Bat-eared fox	LC	stable	5
Bobcat	LC	stable	6	Bush dog	NT	decreasing	6
Clouded leopard	VU	decreasing	4	Grey fox	LC	stable	6
Sunda clouded leopard	VU	decreasing	3	Island fox	NT	increasing	1
Manul	NT	decreasing	4	Bengal fox	LC	decreasing	4
Lion	VU	decreasing	4	Blandford׳s fox	LC	stable	4
Jaguar	NT	decreasing	5	Cape fox	LC	stable	4
Leopard	VU	decreasing	5	Corsac fox	LC	unknown	5
Tiger	EN	decreasing	3	Tibetan fox	LC	unknown	4
Snow leopard	EN	decreasing	3	Arctic fox	LC	stable	6
Marbled cat	NT	decreasing	4	Kit fox	LC	decreasing	4
Leopard cat	LC	stable	5	Pallid fox	LC	unknown	4
Flat-headed cat	EN	decreasing	2	Rüppell׳s fox	LC	stable	6
Rusty-spotted cat	NT	decreasing	4	Swift fox	LC	stable	3
Fishing cat	VU	decreasing	3	Red fox	LC	stable	7
Puma	LC	decreasing	7	Fennec fox	LC	stable	6
Jaguarundi	LC	decreasing	6

**Fig. 3:**
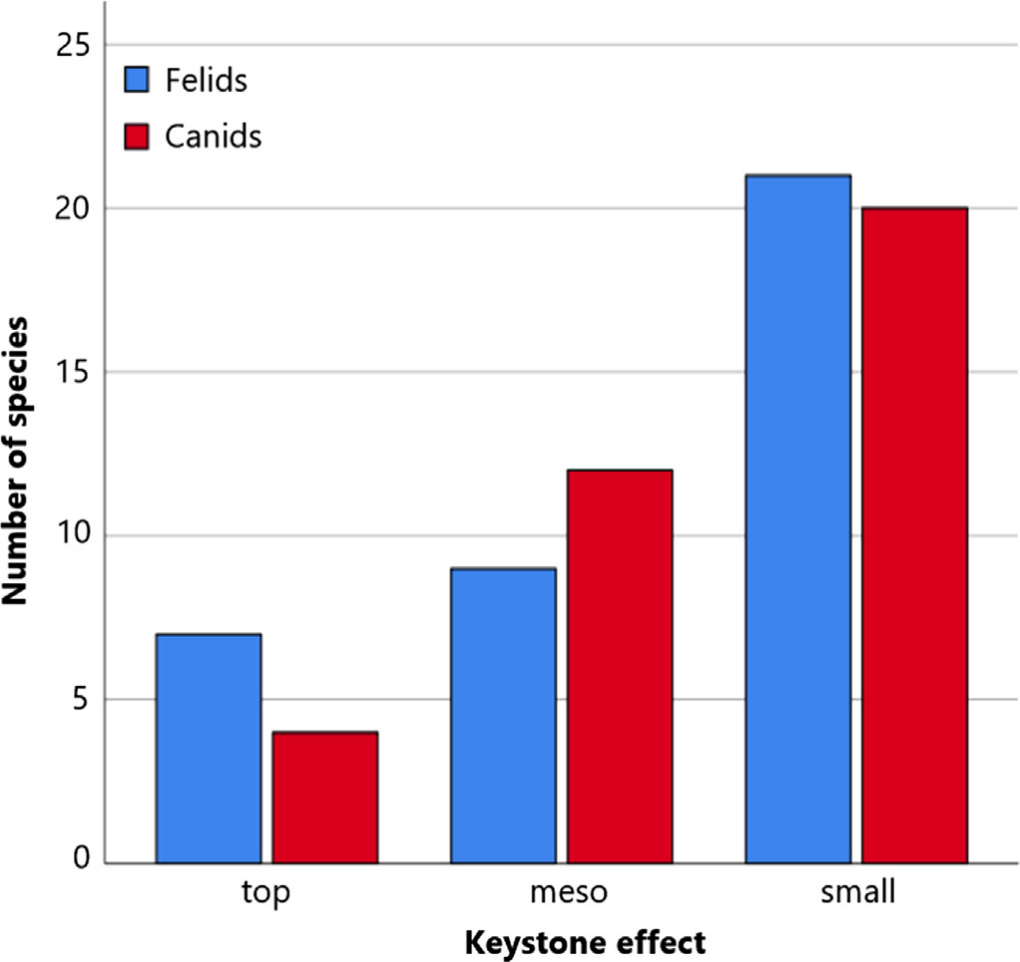
Keystone effect of felid and canid species. Species are either a (1) top predator with a strong top-down effect in a functional group, (2) meso predator with a moderate top-down effect in a functional group, or (3) small predator with a minor top-down effect in a functional group.

The likelihood of being a keystone species (hereafter keystone effect) was predicted for each species and based on the following definition: “a strongly interacting species whose top-down effect on species diversity and competition is large relative to its biomass dominance within a functional group [11].” The keystone effect was divided into three categories: (1) top predator with a strong top-down effect in a functional group, (2) meso predator with a moderate top-down effect in a functional group, and (3) small predator with a minor top-down effect in a functional group (Table 3). The majority of felid and canid species are small predators (Fig. 3).

**Table 3:** Felid and canid species listed into three categories: (1) top predator with a strong top-down effect in a functional group, (2) meso predator with a moderate top-down effect in a functional group, and (3) small predator with a minor top-down effect in a functional group.

**Keystone effect**	*Felidae species*	*Canidae species*
Category 1	Canada lynx	Coyote
	Lion	Grey wolf
	Jaguar	Red wolf
	Leopard	Ethiopian wolf
	Tiger	
	Snow leopard	
	Puma	
Category 2	Cheetah	Side-striped jackal
	Caracal	African golden wolf
	Ocelot	Golden jackal
	Serval	Black-backed jackal
	Eurasian lynx	Crab-eating fox
	Iberian lynx	Maned wolf
	Bobcat	Dhole
	Clouded leopard	African wild dog
	Sunda clouded leopard	Raccoon dog
		Island fox
		Arctic fox
		Red fox
Category 3	African golden cat	Short eared dog
	Bay cat	Culpeo
	Asiatic golden cat	Darwin׳s fox
	Jungle cat	South American gray fox
	Sand cat	Pampas fox
	Black footed cat	Sechura fox
	Wild cat	Hoary fox
	Southern tigrina	Bat-eared fox
	Oncilla	Bush dog
	Margay	Grey fox
	Pampas cat	Bengal fox
	Geoffroy׳s cat	Blandford׳s fox
	Kodkod	Cape fox
	Andean mountain cat	Corsac fox
	Manul	Tibetan fox
	Marbled cat	Kit fox
	Leopard cat	Pallid fox
	Flat-headed cat	Rüppell׳s fox
	Rusty-spotted cat	Swift fox
	Fishing cat	Fennec fox
	Jaguarundi

Taxonomic uniqueness was listed for each species, by deriving Evolutionary Distinctiveness (ED) scores and Evolutionary Distinct and Globally Endangered (EDGE) scores [12]; the higher the score, the higher a species’ conservation priority (Table 4). We also predicted taxonomic uniqueness by counting the number of species per genus; a monotypic genus, which consists of only one representative, has a higher conservation priority Table 5.

**Table 4:** Evolutionary Distinctiveness (ED) and Evolutionary Distinct and Globally Endangered (EDGE) scores, and the number of species per genus for felid and canid species.

**Felidae species**				**Canidae species**			
**Species name**	**ED score**	**EDGE score**	**No. in genus**	**Species name**	**ED score**	**EDGE score**	**No. in genus**
Cheetah	13.45	4.1	1	Short eared dog	3.69	2.24	1
African golden cat	9.32	3.03	2	Side-striped jackal	3.46	1.49	8
Caracal	9.77	2.38	2	African golden wolf			8
Bay cat	9.11	4.39	2	Golden jackal	3.46	1.49	8
Asiatic golden cat	9.11	3.01	2	Coyote	3.25	1.45	8
Jungle cat	7.37	2.12	4	Grey wolf	3.26	1.45	8
Sand cat	7.54	2.84	4	Black-backed jackal	3.56	1.52	8
Black footed cat	7.55	3.53	4	Red wolf			8
Wild cat	7.27	2.11	4	Ethiopian wolf	3.22	3.52	8
Ocelot	8.94	2.3	8	Crab-eating fox	3.86	1.58	1
Southern tigrina			8	Maned wolf	3.78	2.26	1
Oncilla	8.13	3.6	8	Dhole	3.79	3.65	1
Margay	8.94	2.99	8	Culpeo	2.74	1.32	6
Pampas cat	7.34	2.82	8	Darwin׳s fox	2.82	4.11	6
Geoffroy׳s cat	8.23	2.92	8	South American gray fox	2.82	1.34	6
Kodkod	8.16	3.6	8	Pampas fox	2.74	1.32	6
Andean mountain cat	8.15	4.29	8	Sechura fox	2.73	2.01	6
Serval	9.84	2.38	1	Hoary fox	3.01	1.39	6
Canada lynx	7.97	2.19	4	African wild dog	3.87	3.66	1
Eurasian lynx	7.98	2.2	4	Raccoon dog	7.92	2.19	1
Iberian lynx	8.44	5.02	4	Bat-eared fox	8.49	2.25	1
Bobcat	9.61	2.36	4	Bush dog	3.7	2.24	1
Clouded leopard	7.28	3.5	2	Grey fox	6.4	2	2
Sunda clouded leopard	7.28	3.5	2	Island fox	6.4	2.69	2
Manul	8.99	2.99	1	Bengal fox	5.42	1.86	12
Lion	8.26	3.61	5	Blandford׳s fox	4.53	1.71	12
Jaguar	8.29	2.92	5	Cape fox	5.44	1.86	12
Leopard	8.27	2.92	5	Corsac fox	3.48	1.5	12
Tiger	8.33	4.31	5	Tibetan fox	3.48	1.5	12
Snow leopard	8.38	4.32	5	Arctic fox	3.8	1.57	12
Marbled cat	9.23	3.71	1	Kit fox	3.5	1.5	12
Leopard cat	9.89	2.39	4	Pallid fox	5.51	1.87	12
Flat-headed cat	10.69	4.54	4	Rüppell׳s fox	3.48	1.49	12
Rusty-spotted cat	9.97	3.78	4	Swift fox	3.5	1.5	12
Fishing cat	9.88	4.47	4	Red fox	3.5	1.49	12
Puma	11.89	2.56	2	Fennec fox	4.53	1.71	12
Jaguarundi	11.93	2.56	2

**Table 5:** All scientific articles published on felid and canid species between 2013 and 2017. Research topics are (1) ecology and behaviour, (2) conservation and wildlife management, (3) anatomy and physiology, (4) diseases and other health issues, (5) captive housing and artificial reproduction, (6) genetic diversity and phylogenetic structure, or (7) taxonomy and palaeoecology.

	**Articles**	**Research topics**						
	*Total*	1	2	3	4	5	6	7
*Felidae species*								
Cheetah	161	36	27	12	57	24	5	0
African golden cat	3	1	2	0	0	0	0	0
Caracal	10	6	1	2	0	1		0
Bay cat	2	0	1	0	0	0	0	1
Asiatic golden cat	7	2	1	0	0	1	1	2
Jungle cat	4	0	1	0	1	1	1	0
Sand cat	6	1	2	1	1	0	1	0
Black footed cat	6	1	0	1	3	1	0	0
Wild cat	60	14	8	3	17	4	14	0
Ocelot	59	27	9	4	11	3	5	0
Southern tigrina	7	3	0	1	0	1	1	1
Oncilla	12	4	0	1	3	2	1	1
Margay	10	6	1	1	2	0	0	0
Pampas cat	6	1	1	1	1	0	2	0
Geoffroy׳s cat	13	5	3	0	0	0	4	1
Kodkod	14	5	4	0	1	1	3	0
Andean mountain cat	3	0	3	0	0	0	0	0
Serval	7	4	0	0	3	0	0	0
Canada lynx	48	21	13	0	5	2	7	0
Eurasian lynx	118	41	33	4	13	17	7	3
Iberian lynx	51	9	9	0	13	9	5	6
Bobcat	96	30	25	3	32	0	6	0
Clouded leopard	24	6	4	2	4	6	0	2
Sunda clouded leopard	10	4	6	0	0	0	0	0
Manul	7	0	0	0	5	2	0	0
Lion	278	59	102	14	57	21	16	9
Jaguar	164	41	75	4	20	9	10	5
Leopard	232	61	99	15	20	9	18	10
Tiger	359	44	157	24	66	28	37	3
Snow leopard	80	18	44	2	6	3	6	1
Marbled cat	4	1	3	0	0	0	0	0
Leopard cat	45	13	11	1	12	1	6	1
Flat-headed cat	2	0	1	0	0	0	1	0
Rusty-spotted cat	1	0	0	0	0	1	0	0
Fishing cat	10	0	5	1	2	2	0	0
Puma	276	108	103	10	28	6	17	4
Jaguarundi	10	4	0	1	4	0	1	0
**Total**	**2205**	**576**	**754**	**108**	**387**	**155**	**170**	**50**
*Canidae species*								
Short eared dog	0	0	0	0	0	0	0	0
Side-striped jackal	0	0	0	0	0	0	0	0
African golden wolf	6	2	2	0	0	0	1	1
Golden jackal	101	32	13	11	33	0	10	2
Coyote	228	89	76	6	34	7	15	1
Grey wolf	597	175	198	21	80	10	92	21
Black-backed jackal	22	10	3	2	4	1	2	0
Red wolf	36	6	15	2	7	3	2	1
Ethiopian wolf	16	5	3	0	7	0	1	0
Crab-eating fox	61	7	2	17	30	3	2	0
Maned wolf	52	1	10	9	22	4	5	1
Dhole	33	13	11	3	2	1	0	3
Culpeo	14	6	3	3	2	0	0	0
Darwin׳s fox	3	0	0	0	2	0	1	0
South American gray fox	9	1	2	1	4	0	0	1
Pampas fox	21	2	1	6	10	0	2	0
Sechura fox	1	0	1	0	0	0	0	0
Hoary fox	6	0	0	1	5	0	0	0
African wild dog	74	24	31	2	5	6	4	2
Raccoon dog	148	11	12	23	70	6	24	2
Bat-eared fox	13	9	0	2	1	0	0	1
Bush dog	8	1	2	0	3	2	0	0
Grey fox	14	6	1	0	6	0	1	0
Island fox	28	5	12	0	6	1	4	0
Bengal fox	5	1	1	1	2	0	0	0
Blandford׳s fox	1	1	0	0	0	0	0	0
Cape fox	3	3	0	0	0	0	0	0
Corsac fox	7	1	1	1	2	0	1	1
Tibetan fox	8	2	0	0	3	0	2	1
Arctic fox	95	20	4	13	29	10	15	4
Kit fox	26	10	11	1	2	0	2	0
Pallid fox	3	2	0	0	0	0	1	0
Rüppell׳s fox	1	0	0	0	0	0	0	1
Swift fox	14	1	7	0	3	0	3	0
Red fox	485	92	79	23	226	4	55	6
Fennec fox	6	0	0	1	3	0	1	1
**Total**	**2145**	**538**	**501**	**149**	**603**	**58**	**246**	**50**

All scientific articles published on felid and canid species between 2013 and 2017 were listed (Supplementary material S1 for felids and S2 for canids). The research papers were subdivided into the following research topics: (1) ecology and behaviour, (2) conservation and wildlife management, (3) anatomy and physiology, (4) diseases and other health issues, (5) captive housing and artificial reproduction, (6) genetic diversity and phylogenetic structure, and (7) taxonomy and palaeoecology. For felids, most research papers were related to conservation and wildlife management, and for canids most papers were related to diseases and other health issues (Table 5).

## 2 Experimental design, materials and methods

Literature searches were conducted in Scopus, EBSCO and Google Scholar to optimize the yield of scientific articles [13]. Common and scientific species names [10] were used as search strings in the electronic databases, for instance: cheetah OR *Acinoyx jubatus*. All peer-reviewed articles that were published between 2013 and 2017 were included. Subspecies were not investigated separately in this literature search, and domesticated animals were excluded. Observational notes or replies to previous publications were also excluded from the database, as well as articles for which no English abstract was available. Articles were listed for species only if the animal in question was the main research topic or among a maximum of three. The research papers were subdivided into research topics that were created during the literature searches and partly based on previous studies [7], [14]. The data led to an overview of species characteristics and the number of articles published between 2013 and 2017 for felid and canid species. The data can be used to assess potential bias in research and conservation prioritization [1].

## Appendix A Supplementary material

S1. List of articles published between 2013 and 2017 for each felid species (*n* = 37). Supplementary material

S2. List of articles published between 2013 and 2017 for each canid species (*n* = 36). Supplementary material
