# Supplementary material for: Species characteristics of felids and canids, and the number of articles published for each species between 2013 and 2017
Source: Data Brief. 2018 Oct 3;21:201–11. doi: 10.1016/j.dib.2018.09.132 (PMC6197655; doi:10.1016/j.dib.2018.09.132)
Supplement: Supplementary file 1 — Supplementary material [file mmc1.pdf]

Author Declaration of Interest

To whom it may concern,

I wish to confirm that there are no known conflicts of interest associated with this publication and there has been no significant financial support for this work that could have influenced its outcome.

I confirm that the manuscript has been read and approved by all named authors and that there are no other persons who satisfied the criteria for authorship but are not listed. I confirm to have given due consideration to the protection of intellectual property associated with this work and that there are no impediments to publication, including the timing of publication, with respect to intellectual property. In so doing I confirm that I have followed the regulations of our institutions concerning intellectual property.

I understand that the Corresponding Author is the sole contact for the Editorial process (including Editorial Manager and direct communications with the office).

I confirm that I have provided a current, correct email address which is accessible by the Corresponding Author and which has been configured to accept email from.

Signed by the author, Laura Tensen, on 8/8/2018

Email: tensen.laura@gmail.com
Phone: +27 (0)74 622 5598

University of Johannesburg
Cnr Kingsway and University road
Auckland Park Campus
D3 Lab 339, Dept Zoology
South Africa, 2006

Ecological Genomics & Wildlife Conservation
